# The Epithelial to Mesenchymal Transition Promotes Glutamine Independence by Suppressing *GLS2* Expression

**DOI:** 10.3390/cancers11101610

**Published:** 2019-10-22

**Authors:** Esmeralda Ramirez-Peña, James Arnold, Vinita Shivakumar, Robiya Joseph, Geraldine Vidhya Vijay, Petra den Hollander, Neeraja Bhangre, Paul Allegakoen, Rishika Prasad, Zachary Conley, José M. Matés, Javier Márquez, Jeffrey T. Chang, Suhas Vasaikar, Rama Soundararajan, Arun Sreekumar, Sendurai A. Mani

**Affiliations:** 1 National Cancer Institute, Cancer Prevention Fellowship Program, Division of Cancer Prevention, Bethesda, MD 20892, USA; esmeralda.ramirez-pena@nih.gov; 2 Department of Molecular and Cell Biology, Baylor College of Medicine, Houston, TX 77030, USA; j.arnold221@gmail.com (J.A.); Arun.Sreekumar@bcm.edu (A.S.); 3 Wiess School of Natural Sciences, Rice University, Houston, TX 77005, USA; vs28@rice.edu; 4 Department of Translational Molecular Pathology, MD Anderson Cancer Center, Houston, TX 77030, USA; RJoseph7@mdanderson.org (R.J.); PDHollander@mdanderson.org (P.d.H.); rprasad3@mdanderson.org (R.P.); SVVasaikar@mdanderson.org (S.V.); RSoundararajan@mdanderson.org (R.S.); 5 Department of Immunology, Mayo Clinic, Jacksonville, FL 32224, USA; Raja.geraldine@mayo.edu; 6 Department of Fibrosis Biology, Gilead Sciences, Foster City, CA 94404, USA; neeraja.bhangre@gilead.com; 7 Department of Medicine, University of California-San Francisco, San Francisco, CA 94143, USA; paul.allegakoen@ucsf.edu; 8 Center for Science Outreach, Department of Teaching and Learning, Vanderbilt University, Nashville, TN 37235, USA; zachary.c.conley@vanderbilt.edu; 9 Canceromics Lab, Department of Molecular Biology and Biochemistry, University of Málaga and Instituto de Investigación Biomedica de Málaga (IBIMA), 29071 Málaga, Spain; jmates@uma.es (J.M.M.); marquez@uma.es (J.M.); 10 Department of Integrative Biology and Pharmacology, University of Texas Health Science Center, Houston, TX 77030, USA; Jeffrey.T.Chang@uth.tmc.edu

**Keywords:** *GLS2*, EMT, glutamine metabolism, breast cancer, *FOXC2*

## Abstract

Identifying bioenergetics that facilitate the epithelial to mesenchymal transition (EMT) in breast cancer cells may uncover targets to treat incurable metastatic disease. Metastasis is the number one cause of cancer-related deaths; therefore, it is urgent to identify new treatment strategies to prevent the initiation of metastasis. To characterize the bioenergetics of EMT, we compared metabolic activities and gene expression in cells induced to differentiate into the mesenchymal state with their epithelial counterparts. We found that levels of *GLS2*, which encodes a glutaminase, are inversely associated with EMT. *GLS2* down-regulation was correlated with reduced mitochondrial activity and glutamine independence even in low-glucose conditions. Restoration of *GLS2* expression in *GLS2*-negative breast cancer cells rescued mitochondrial activity, enhanced glutamine utilization, and inhibited stem-cell properties. Additionally, inhibition of expression of the transcription factor FOXC2, a critical regulator of EMT in *GLS2*-negative cells, restored GLS2 expression and glutamine utilization. Furthermore, in breast cancer patients, high *GLS2* expression is associated with improved survival. These findings suggest that epithelial cancer cells rely on glutamine and that cells induced to undergo EMT become glutamine independent. Moreover, the inhibition of EMT leads to a GLS2-directed metabolic shift in mesenchymal cancer cells, which may make these cells susceptible to chemotherapies.

## 1. Introduction

Highly proliferative tumors consume glucose and amino acids, including glutamine, to sustain the metabolic demands that support biomass production [1]. Glutamine is a conditionally essential amino acid that can be obtained from the microenvironment or synthesized de novo. Glutaminolysis is the process whereby glutamine is converted to glutamate and then to α-ketoglutarate, which enters the tricarboxylic acid (TCA) cycle. The TCA cycle supports oxidative phosphorylation and energy generation and provides a carbon source for fatty acid synthesis, a nitrogen source for synthesis of amino acids and nucleotides, and intermediates necessary for the synthesis of reduced glutathione (GSH), which neutralizes reactive oxygen species [2].

In mammals, including humans, two enzymes, GLS and GLS2, catalyze the conversion of glutamine to glutamate. GLS and GLS2 have distinct patterns of expression and regulation in different organs and tumor types [3]. GLS is ubiquitously expressed and is also highly expressed in several types of tumors as a result of direct regulation by oncogenes such as KRAS and MYC [4,5]. GLS2 is expressed mostly in the liver, brain, and pancreas and is directly regulated by p53, p63, and p73 [6,7,8]. In glioblastoma and cancers of liver and colon, GLS2 expression is lost due to DNA methylation of the *GLS2* gene [9,10].

Many tumors depend on glutamine for growth, and glutamine addiction is associated with GLS [4,11]. Pharmacological agents that target GLS directly, such as CB-839, are currently in clinical trials [12]. Although glutamine addiction has been observed in many cancers, recent studies employing three-dimensional organoid cultures and in vivo models using fluorinated glutamine have demonstrated that not all tumor types metabolize glutamine [13]. The observed glutamine independence of some tumors could confer resistance to glutaminase inhibitors [14]. The contribution of GLS2 to glutamine dependence in these tumors has not been examined. 

Considerable evidence suggests that the epithelial to mesenchymal transition (EMT) program contributes to the development of therapy resistance and metastasis [15,16,17,18,19,20,21,22,23,24]. We have previously demonstrated that EMT promotes acquisition of stem-cell properties by cancer cells [25,26]. In this study, we found that the induction of EMT results in the suppression of *GLS2* expression and the promotion of glutamine independence even in low-glucose conditions and in the presence of GLS. In addition, we observed that GLS2 re-expression enhanced glutamine consumption and reduced sphere formation. The transcription factor FOXC2 is critical to maintaining mesenchymal and stem-cell properties [27,28] and has been shown to direct metabolic activities in adipocytes [29,30,31,32,33,34,35,36,37,38]. We found that inhibition of FOXC2 expression (and thus inhibition of EMT) also restored GLS2 expression and glutamine dependency in cells that had undergone EMT. We evaluated *GLS2* expression in breast cancer patients and found that, in line with our data, high *GLS2* expression is inversely correlated with the EMT gene signature. Further, we found that *GLS2* copy number deletions were over-represented in the basal breast cancer subtype; a subtype with poor clinical outcomes and high metastatic potential [39]. In support of the idea that tumor cells with high GLS2 expression have less aggressive characteristics, we found that high *GLS2* expression correlates with improved overall survival in breast cancer patients.

## 2. Results

### 2.1. GLS2 Expression Is Inversely Correlated with EMT in Breast Cancer

**Figure 1:**
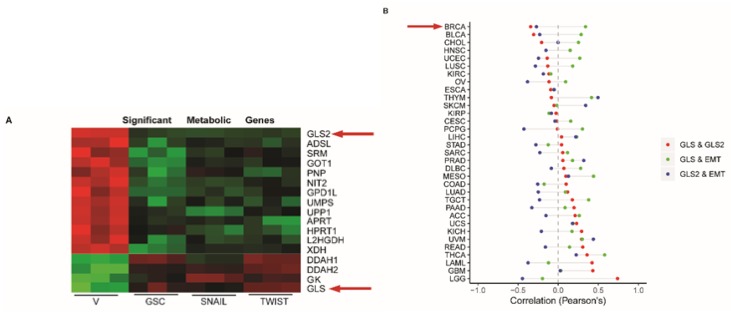
*GLS2* is inversely correlated with epithelial to mesenchymal transition (EMT) in breast cancer patients. (**A**) Heatmap of mRNA expression of metabolism-associated genes obtained from a previously reported analysis [26] of HMLE cells treated with vector only (V) and in HMLE cells that express GSC, Snail, or Twist. *GLS2* and *GLS* are indicated by arrows. (**B**) Plots of correlation between *GLS* and *GLS2* (red circles), *GLS* and an EMT gene signature (green circles), and *GLS2* and an EMT signature (blue circles) in difference cancer types. The breast cancer tumor (BRCA) relationship is indicated by the arrow.

To identify metabolic genes and pathways that are specifically altered in cells induced to undergo EMT relative to epithelial counterparts, we analyzed the expression of metabolic genes from EMT gene expression data previously published by our lab [26]. For this analysis we compared HMLE cell lines, which are immortalized human mammary epithelial cells, engineered to express EMT-inducing transcription factors Goosecoid (HMLE-GSC), Snail (HMLE-Snail), and Twist (HMLE-Twist) with vector control (HMLE-V) cells. In cells that had undergone EMT, *GLS* was induced and *GLS2* was suppressed compared to control epithelial cells, even though both have the capacity to convert glutamine to glutamate (Figure 1A). We evaluated GLS2 and GLS expression levels in additional cell lines and found that GLS2 expression was reduced in mesenchymal breast cancer cell lines (e.g., SUM159, MDA231, and MDA 468) relative to the epithelial breast cancer cell line (MCF7) and that GLS expression was enhanced (Supplementary Figure S1A–C). It was previously reported that in a model of EMT induced by treating non-transformed mammary epithelial MCF10A cells with TGFβ1, *GLS* expression is enhanced compared to cells treated with vehicle control [40]. In this model, we found that *GLS2* expression is suppressed (Supplementary Figure S1D). In agreement with the previous study, *GLS* expression was induced following the exposure to TGFβ1 (Supplementary Figure S1E).

To determine if the GLS2 and GLS inverse expression pattern we observed in the cell lines is also evident in breast cancer patient samples, we compared *GLS* and *GLS2* copy numbers in samples from 1075 patients using data from The Cancer Genome Atlas (TCGA). In this analysis we observed that the 143 patients who had lost one copy of *GLS2* exhibited a corresponding reduction in *GLS2* RNA expression (Supplementary Figure S1F). Notably, when we compared amplifications and deletions of GLS2 among the PAM50 subtypes, we observed that the majority of the *GLS2* copy number deletions occurred in the mesenchymal stem cell-rich basal-like breast cancer subtype (Supplementary Figure S1G). Furthermore, we found mostly *GLS* copy number amplifications were also observed in this subtype (Supplementary Figure S1G).

From the TCGA, data we also examined the expression of *GLS2* in more than 11,000 patients with 33 different cancer types. We compared *GLS2* expression with *GLS* expression as well as with expression of EMT signature genes (Hallmarks, MSigdb v6). In line with results obtained from analysis of cell lines, expression of *GLS2* is inversely correlated with *GLS* in 52% of the cancer types analyzed, including breast cancer (Figure 1B). We also found that the expression of *GLS2* is inversely correlated with the EMT signature gene expression in 70% of the cancer types analyzed (Figure 1B). These findings suggest that the inverse correlation of *GLS2* with *GLS* and with EMT signature genes is highly orchestrated.

### 2.2. Cells that Undergo the Epithelial to Mesenchymal Transition Fail to Utilize Glutamine

**Figure 2:**
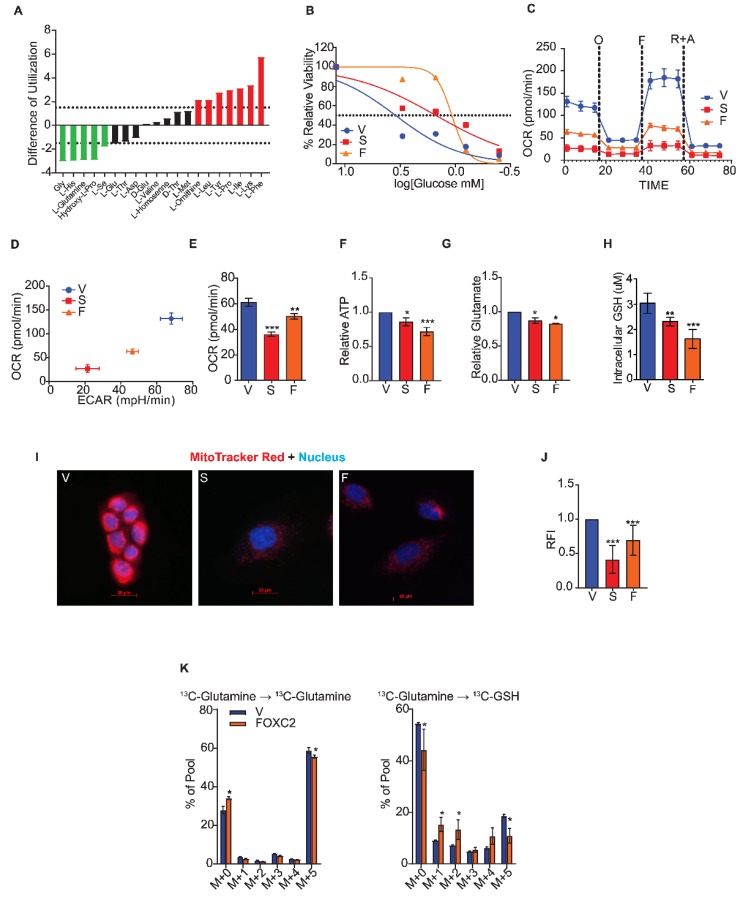
Cells that undergo EMT fail to utilize glutamine. (**A**) The difference in amino acid utilization between HMLER cells that express Snail and control cells that express vector only at 8 h. Negative values indicate metabolic loss (green). Positive values indicate metabolic gain (red). Dotted lines at -1.5 and 1.5 indicate significance. (**B**) Viability of HMLER cells that express vector only (V), Snail (S), and FOXC2 (F) cells after 24 h in 0, 0.4, 0.8, 1.5, 3, and 12 mM glucose quantified relative to viability in normal growth media using MTS assay. (**C**) Oxygen consumption of control cells (V, *n* = 6) and cells that express Snail (S, *n* = 5) and FOXC2 (F, *n* = 5) over time (in minutes) after addition of oligomycin (O), FCCP (F), and rotenone plus antimycin (R + A) measured using a Seahorse Analyzer. (**D**) OCR versus ECAR measured using Seahorse Analyzer in control (V, *n* = 6), Snail-expressing (S, *n* = 5), and FOXC2-expressing (F, *n* = 5) HMLER cells. (**E**) Basal respiration rate (pmol/min) of HMLER control cells (V, *n* = 6) and cells that express Snail (S, *n* = 5) and FOXC2 (F, *n* = 5) as measured using a Seahorse XFe96 Analyzer. (**F**) ATP production measured by luminescence in Snail-expressing (S, *n* = 5) and FOXC2-expressing (F, *n* = 5) HMLER cells relative to production by control cells (V, *n* = 5). (**G**) Quantification of relative intracellular glutamate in Snail-expressing (S, *n* = 3) and FOXC2-expressing (F, *n* = 3) cells relative to control (V, *n* = 3) cells. (**H**) Quantification of intracellular GSH in control (V, *n* = 5), Snail-expressing (*n* = 5), and FOXC2-expressing (*n* = 5) HMLER cells. (**I**) Representative immunofluorescence images of HMLER control (V), Snail-expressing (S), and FOXC2-expressing (F) cells stained with MitoTracker Red (red) and DAPI (blue). Scale bar: 20 µm. (**J**) Relative fluorescence intensity (RFI) of MitoTracker staining in Snail-expressing (S, *n* = 100) and FOXC2-expressing (F, *n* = 100) HMLER cells versus control cells (V, *n* = 100) quantified using ImageJ software. (**K**) U^13^C-glutamine flux in FOXC2-expressing (*n* = 4) and control (V, *n* = 4) HMLER cells determined by mass spectrometry. The data are reported as means ± SD; * *p* ≤ 0.05, ** *p* ≤ 0.01, *** *p* ≤ 0.001, NS indicates *p* > 0.05.

Since we observed increased expression of GLS and decreased expression of GLS2 following the induction of EMT, we hypothesized that the cells induced to undergo EMT would have enhanced glutamine utilization compared to their epithelial counterpart. To examine this idea in an unbiased manner, we investigated the utilization of amino acids, including glutamine, by performing a Biolog phenotypic metabolic microarray assay. This assay measured the utilization of individual amino acids by quantifying color changes in a redox dye during the logarithmic phase of growth over 24 h in Ras-transformed HMLE cells (the HMLER line) induced to undergo EMT by stable expression of the transcription factor Snail relative to the vector-expressing control cells (Figure 2A). The Snail-expressing cells had a 3-fold loss in the ability to metabolize L-glutamine compared to the control cells. This was an unexpected finding considering that GLS levels are high in Snail-expressing HMLER cells.

To further characterize nutrient utilization, we measured glucose utilization and the glycolytic index in cells induced to undergo EMT compared to the control cells (Figure 2C,D). To assess glucose utilization, we measured the concentration of glucose at which the viability of cells was reduced by 50% compared to growth in excess glucose using an MTS assay. These concentrations for HMLER cells that expressed either Snail or FOXC2 were 1.5- and 1.07-mM glucose, respectively, whereas the control cell growth was reduced by half at 3.47 mM (Figure 2B). Thus, the cells that had undergone EMT were less dependent on glucose than epithelial-like controls. By measuring the extracellular acidification rate (ECAR) and the oxygen consumption rate (OCR) using the Seahorse assay, we found that cells that have undergone EMT have a higher glycolytic capacity than do the control epithelial cells (Supplementary Figure S2A). To further assess the respiration of cells that have undergone EMT, we measured OCR over time using the Seahorse MitoStress assay. Cells induced to undergo EMT had lower spare respiratory capacity in response to the mitochondrial complex inhibitors oligomycin, carbonyl cyanide p-[trifluoromethoxy]-phenyl-hydrazone, and rotenone plus antimycin than the control cells (Figure 2C), as shown by lower OCR/ECAR ratios (Figure 2D). Moreover, cells induced to undergo EMT had significantly lower basal respiration (Figure 2E) and lower ATP production (Figure 2F). To characterize the intracellular metabolites in cells that have undergone EMT, we performed a steady-state mass spectrometry analysis and found that glycolysis metabolites 3PG/2PG, FBP/GBP, G6P/F6P, and PEP were significantly more abundant in HMLER cells that expressed FOXC2 than in control cells (Supplementary Figure S2B).

Since cells induced to undergo EMT have lower mitochondrial activity than control epithelial cells and fail to utilize glutamine, we hypothesized that the conversion of glutamine to glutamate is impaired after EMT, resulting in lower production of GSH. To test this, we measured the biochemical conversion of intracellular glutamine to glutamate in cells induced to undergo EMT and control epithelial cells. The HMLER cells that expressed Snail or FOXC2 converted glutamine to glutamate significantly less efficiently than control epithelial cells even though the cells that had undergone EMT expressed a substantially higher level of GLS (Figure 2G). We also detected a reduction of intracellular GSH, the byproduct of glutamine metabolism, in the cells that had undergone EMT compared to control cells (Figure 2H). In addition, we found that cells that expressed Snail or FOXC2 had lower mitochondrial membrane potential as evidenced by the reduced staining with MitoTracker Red dye compared to control cells (Figure 2I,J). To characterize the breakdown of glutamine and conversion into GSH, we performed a mass spectrometry-based flux analysis and found 2-fold lower incorporation of ^13^C into GSH in HMLER cells that expressed FOXC2 than control cells after 6 h of growth in the presence of the ^13^C-labeled glutamine (Figure 2K). 

We also found that the mesenchymal breast cancer cell lines (SUM159 and MDA-MB-231) displayed reduced mitochondrial membrane potential (Supplementary Figure S2C,D), impaired conversion of glutamine to glutamate (Supplementary Figure S2E), and lower levels of reduced GSH (Supplementary Figure S2F) compared to epithelial breast cancer cell line (MCF7). Overall, these findings suggest that the cells that have undergone EMT fail to utilize glutamine, even though they express a high level of *GLS* relative to *GLS2*. Further, our data indicate that induction of EMT results in reduced mitochondrial activity and glutamine independence due to the reduced expression of *GLS2.*

### 2.3. GLS2 Over-Expression Rescues Glutamine Utilization

**Figure 3:**
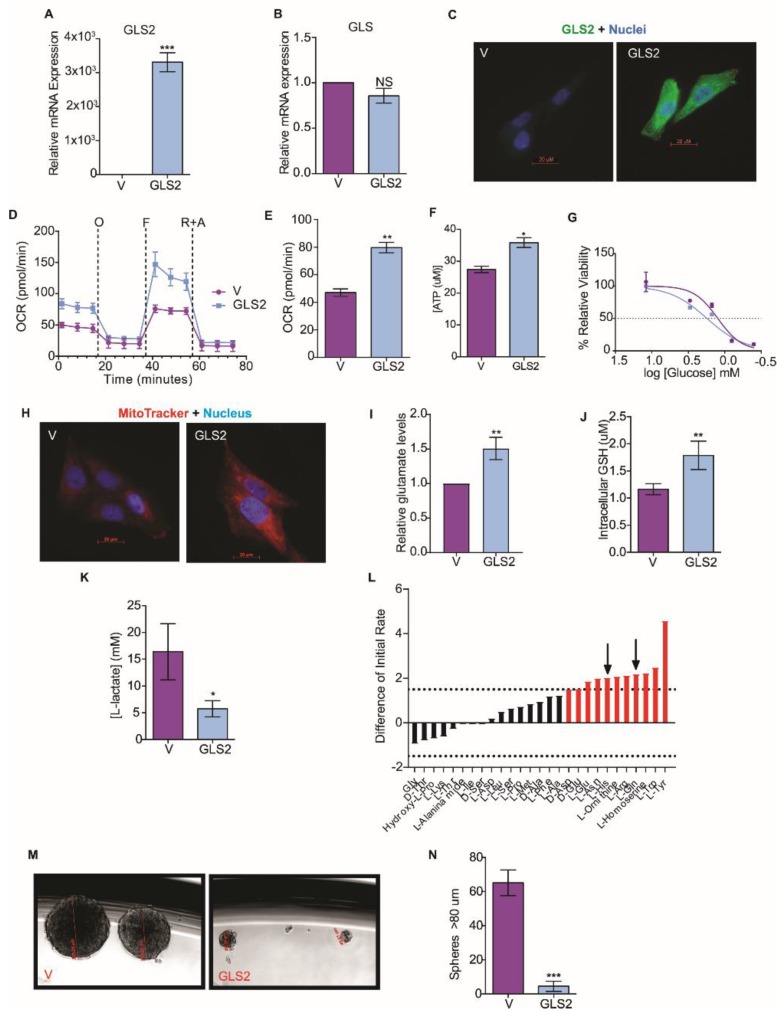
GLS2 over-expression rescues glutamine utilization. (**A**) RT-PCR quantification of *GLS2* mRNA in SUM159 cells that over-express GLS2 (GLS2, *n* = 3) relative to control SUM159 cells (V, *n* = 3). (**B**) RT-PCR quantification of *GLS* mRNA in SUM159 cells that over-express GLS2 (GLS2, *n* = 3) relative to control cells (V, *n* = 3). (**C**) Representative images of control SUM159 cells (V) and SUM159 cells that over-express GLS2 (GLS2) stained with an antibody to GLS2 (green) and with DAPI (blue). scale bar: 20 µm. (**D**) OCR (pmol/min) of control (V, *n* = 5) and SUM159 cells that overexpress GLS2 (GLS2, *n* = 5) over time after addition of oligomycin (O), FCCP (F), and rotenone plus antimycin (R + A) measured using the Seahorse XFe96 Analyzer. (**E**) Basal respiration (pmol/min) of control (V, *n* = 5) and SUM159 cells that overexpress GLS2 (GLS2, *n* = 5) measured using the Seahorse XFe96 Analyzer. (**F**) ATP production (µM) measured by luminescence in SUM159 control (*n* = 5) and GLS2-expressing (*n* = 5) cells. (**G**) Viability of control (V, *n* = 4) and SUM159 cells that overexpress GLS2 (GLS2, *n* = 4) after 24 h in 0, 0.4, 0.8, 1.5, 3, 12 mM quantified relative to viability in normal growth media using the MTS assay. (**H**) Representative images of SUM159 control cells (V) and cells that overexpress GLS2 (GLS2) stained with MitoTracker Red (red) and DAPI (blue). scale bar: 20 µm. (**I**) Quantification of relative intracellular glutamate levels in SUM159 control cells (V, *n* = 3) and SUM159 cells that overexpress GLS2 (GSL2, *n* = 3). (**J**) Quantification of intracellular GSH in SUM159 control (V, *n* = 4) and GLS2 over-expressing cells (GSL2, *n* = 4). (**K**) Quantification of secreted L-lactate in the culture media of SUM159 control (V, *n* = 3) and GLS2 over-expressing cells (GLS2, *n* = 3). (**L**) The difference in amino acid utilization between HMLER cells that overexpress only FOXC2 and that overexpress both FOXC2 and GLS2 at 8 h. Negative values indicate metabolic loss (green bars). Positive values indicate metabolic gain (red bars). Dotted lines at −1.5 and 1.5 indicate significance in loss and gain, respectively. Black arrows indicate the amino acids with restored utilization due to GLS2 expression determined by comparison to Figure 2A. (**M**) Representative phase images of mammospheres greater after 14 days in culture. Scale Bar: 255-49 µm. (**N**) Quantification of mammospheres greater than 80 µm after 14 days in culture. Plotted are means ± SD; * *p* ≤ 0.05, ** *p* ≤ 0.01, *** *p* ≤ 0.001, NS indicates *p* > 0.05.

To understand how *GLS2* suppression metabolically supports the EMT, we overexpressed *GLS2* in the SUM159 breast cancer cell line, a line that has mesenchymal characteristics; experiments were also performed with the same cells expressing control vector (Figure 3A). The overexpression of *GLS2* in these cells did not significantly change the expression of endogenous *GLS* (Figure 3B), *FOXC2* (Supplementary Figure S3A), or *ZEB1* (Supplementary Figure S3B). We observed a similar pattern in Snail and GLS2 expressing HMLER cells (Supplementary Figure S3C,D). GLS2 protein was present in the SUM159 cells transfected with the GLS2 over-expression vector but not in control cells (Figure 3C). SUM159 that express GLS2 had an increased respiratory capacity (Figure 3D), higher basal respiration (Figure 3E), and increased ATP synthesis (Figure 3F) relative to SUM159 vector control cells. GLS2 over-expression in SUM159 cells also resulted in a reduced efficiency of glucose utilization compared to control cells: Viability of cells was reduced by 50% compared to growth in excess glucose at 1.75 mM and 1.25 mM glucose, respectively (Figure 3G). Furthermore, GLS2-overexpressing SUM159 cells had enhanced mitochondrial membrane potential (Figure 3H), enhanced glutaminolysis (Figure 3I), and higher intracellular GSH concentration compared to control cells (Figure 3J). We hypothesize that this shift toward mitochondrial metabolism could decrease glycolytic metabolism, and, indeed, GLS2 over-expressing SUM159 cells secreted significantly lower levels of lactate than the control cells (Figure 3K).

Since GLS2 over-expression in SUM159 cells enhanced glutaminolysis, we examined amino acid utilization in GLS2 over-expressing cancer cells induced to undergo EMT. In this experiment we compared HMLER cells that express FOXC2 and GLS2 to HMLER cells that only express FOXC2: The GLS2-expressing cells more efficiently utilized L-glutamine (Figure 3L). This suggests that the suppression of GLS2 expression results in the inability of cells that have undergone EMT to utilize glutamine.

Previously, we demonstrated that epithelial cells acquire stem-cell properties in response to the induction of EMT [25]. To understand the impact of glutamine metabolism on EMT and stem-cell properties, we measured the induction of EMT markers in SUM159 cells that overexpress GLS2 and found that GLS2 did not reduce the expression of any mesenchymal markers (Supplementary Figure S4). However, GLS2 expression inhibited mammosphere formation by SUM159 cells that overexpress GLS2 (Figure 3M,N), and mammosphere formation is correlated with cancer-stem-cell potential [41]. On average, 7.5 spheres were formed in SUM159-GLS2 cell cultures compared to an average of 65 spheres in SUM159 control cells (Figure 3N). GLS2 expression did not, however, affect proliferation of these cells (Supplementary Figure S3E). Collectively, these data demonstrate that by re-activating a key component that supports glutamine metabolism in cells with EMT properties, a component of cancer-stem-cell potential is inhibited.

### 2.4. Inhibition of FOXC2 Enhances GLS2 Expression and Glutamine Utilization

**Figure 4:**
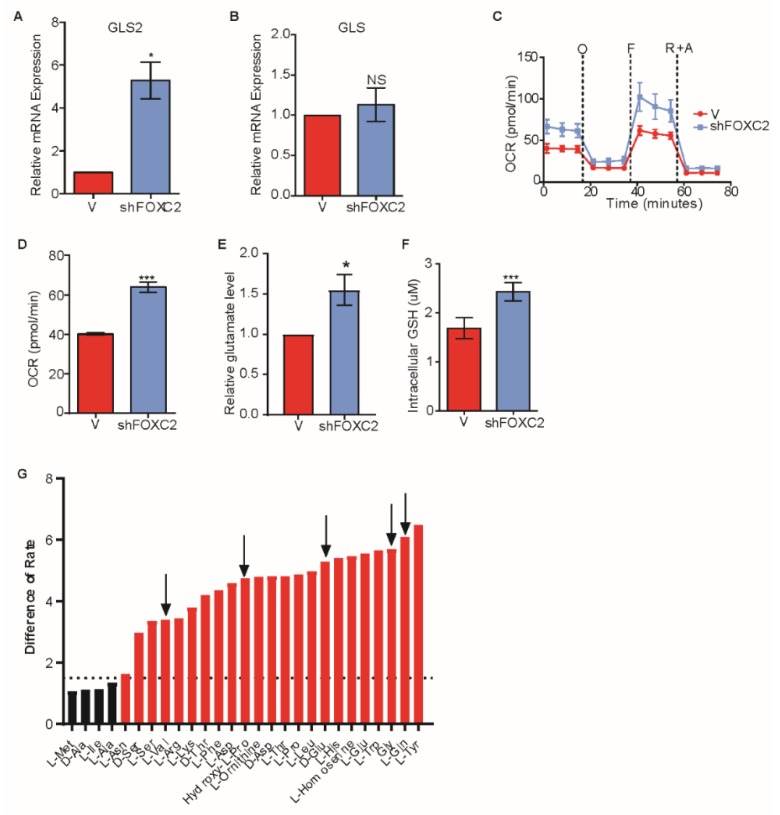
Inhibition of FOXC2 enhances GLS2 expression and glutamine utilization. (**A**,**B**) RT-PCR analysis of A) *GLS2* mRNA and B) *GLS* mRNA in HMLER cells that express Snail without (−) or with (+shFOXC2) expression of shFOXC2. (**C**) OCR of HMLER cells that express Snail without (−, *n* = 6) or with (+shFOXC2, *n* = 6) shFOXC2 over time (in minutes) after addition of oligomycin (O), FCCP (F), and rotenone and antimycin (R + A). (**D**) Basal respiration rate (pmol/min) of HMLER cells that express Snail without (−, *n* = 6) or with (+shFOXC2, *n* = 6) shFOXC2. (**E**) Quantification of intracellular glutamate per 1 × 10^3^ HMLER cells that express Snail without (−, *n* = 3) or with (+shFOXC2, *n* = 3) shFOXC2. (**F**) Quantification of intracellular GSH in HMLER cells that express Snail without (−, *n* = 4) or with (+ shFOXC2, *n* = 5) shFOXC2. (**G**) Difference in utilization between HMLER cells that express Snail without (−, *n* = 3) or with (+shFOXC2, *n* = 3) shFOXC2 at 8 h. Negative values indicate metabolic loss. Positive values indicate metabolic gain. Dotted lines at −1.5 and 1.5 are designated cutoffs to indicate significance in loss (green bars) and gain (red bars). Black arrows point out the amino acids restored from Figure 2A. Plotted are means ± SD; * *p* ≤ 0.05, *** *p* ≤ 0.001, NS indicates *p* > 0.05.

We next sought to determine if inhibiting EMT would result in the induction of GLS2 in cells that express an EMT-inducing transcription factor. We previously reported that transcription factor FOXC2 functions downstream of multiple EMT-inducing signaling pathways and is necessary for EMT [15,42]. We also found that the p38MAPK phosphorylates FOXC2 and inhibition of p38MAPK inhibits FOXC2 expression as well as EMT [28]. Therefore, we analyzed the expression of GLS2 in HMLER cells that stably express Snail following the inhibition of FOXC2 expression using either shRNA (Figure 4A and Supplementary Figure S5A) or p38MAPK inhibitor (Supplementary Figure S5B). We found that the inhibition of EMT resulted in expression of *GLS2* (Figure 4A and Supplementary Figure S5B), but it did not affect the expression of GLS (Figure 4B and Supplementary Figure 4C). The HMLER cells that express Snail also had higher respiratory capacity and basal respiration following the inhibition of FOXC2 expression (Figure 4C,D). Biochemical conversion of glutamine to glutamate in HMLER cells that express Snail was higher with expression of an shRNA targeting *FOXC2* than without (Figure 4E). Furthermore, in accordance with enhanced respiratory capacity, cells that express shFOXC2 had higher levels of GSH than control cells (Figure 4F). These data suggest that blocking EMT through FOXC2 inhibition results in enhanced GLS2 expression and a metabolic shift toward enhanced mitochondrial activity. We also found that inhibiting the EMT restored the ability of cells to utilize glutamine and four other metabolites (Figure 4G) not otherwise utilized by HMLER cells that have undergone EMT (Figure 2A).

### 2.5. GLS2 Expression Correlates with Survival of Breast Cancer Patients

**Figure 5:**
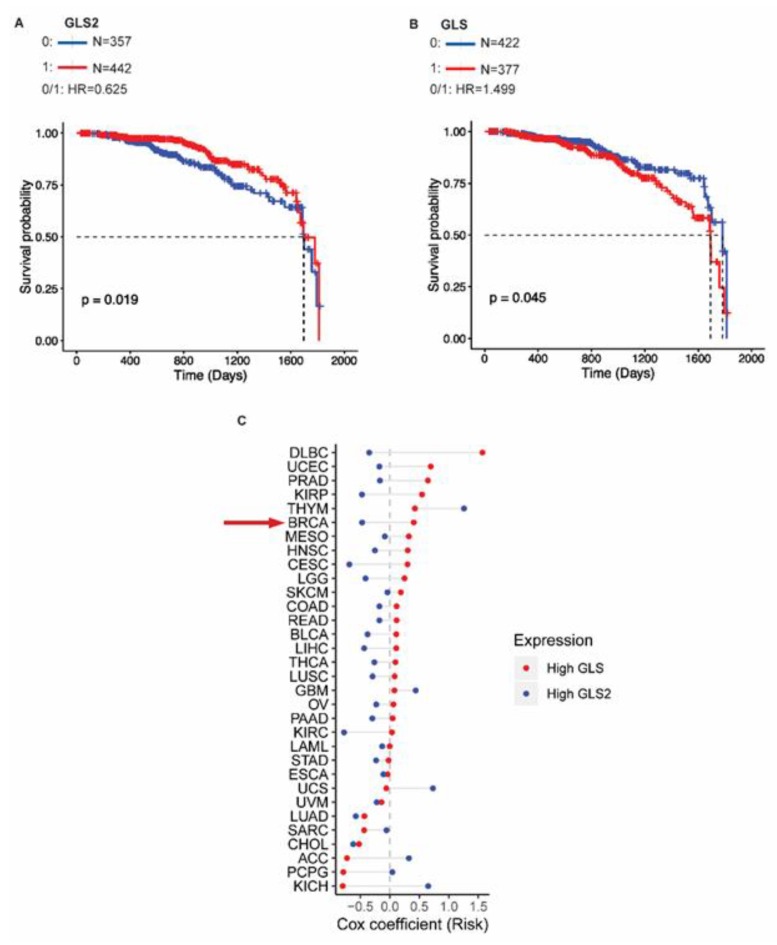
GLS2 expression is associated with better overall survival in breast cancer patients. (**A**) Survival probability versus time for breast cancer patients that express GLS2 (red lines) and those that do not (blue lines). (**B**) Survival probability versus time for breast cancer patients that express GLS (red lines) and those that do not (blue lines). (**C**) Cox risk coefficients for in cancer patients with various types of tumors, including those of the breast (BRCA, indicated with red arrow) associated with expression of GLS (red circle) and GLS2 (blue circle).

To understand how GLS2 expression impacts breast cancer patient outcomes, we analyzed the TCGA data to determine whether *GLS2* expression correlates with survival of breast cancer patients. We found that the absence of *GLS2* expression (blue line) correlates with reduced survival compared to patients whose tumors express *GLS2* (red line) based on a hazard ratio of 0.625 (*p* = 0.019) (Figure 5A). Notably, survival was not significantly correlated with *GLS* expression based on a hazard ratio of 1.499 (*p* = 0.045) (Figure 5B). We then examined the *GLS* and *GLS2* expression patterns and correlation with clinical outcome in 32 different cancer types using TCGA data. For the tumors analyzed, 78% (25 out of 32) expressed *GLS2* and *GLS* in an inverse relationship similar to that observed in breast cancer (Figure 5C). Based on risk coefficients, higher *GLS2* expression (blue circle) relative to *GLS* expression is a predictor of improved survival (risk coefficient < 0) in breast cancer and in 69% (22 out of 32) of the other cancers that we analyzed (Figure 5C). Higher *GLS* expression (red circle) relative to *GLS2* expression correlates with poor survival in 66% (21 out of 32) of the cancers we analyzed (Figure 5C).

## 3. Discussion

We previously identified changes in metabolite abundances, particularly in glutamine and glutamate, that occur with the onset of EMT [43]. Here we demonstrated a striking difference in the expression of two glutaminases, GLS and GLS2, in cells that have and have not undergone EMT. Furthermore, in breast tumors the presence of an EMT signature positively correlates with *GLS* expression and negatively correlates with *GLS2* expression. The highly metastatic basal subtype is enriched with *GLS* amplifications and *GLS2* deletions, but in less aggressive subtypes such as luminal A and luminal B, there are more frequent *GLS2* amplifications and *GLS* deletions. Previous studies have described the opposing roles that GLS and GLS2 have in cancer [44,45,46,47]. For example, the inverse correlation of *GLS* and *GLS2* has been described in the glioblastoma cell line T98G [46,47]. Although both GLS and GLS2 catalyze the conversion of glutamine to glutamate, their localization, expression, and regulation are distinct [3]. *GLS* transcription is directly induced by oncogenes KRAS and MYC, and up-regulation of GLS contributes to glutamine addiction in primary tumors [5,9,10,11,45,46,48].

We sought to understand how the distinct expression patterns of these two glutaminases might metabolically benefit the EMT. We metabolically characterized cells that were induced to undergo EMT and assessed their abilities to utilize glucose and glutamine compared to their epithelial counterparts. We observed that cells that have undergone EMT were viable in low glucose conditions, even in the absence of glutamine, unlike epithelial control cells. We reason that this metabolic flexibility enables cancer cells to survive during the metastatic cascade where they encounter conditions of fluctuating nutrient availability. It has been previously demonstrated that, depending on the substrate used for energy synthesis, some cancer cells acquire metabolic flexibility upon transformation [49]. However, metabolic rigidity can also arise, as in glutamine-dependent cancer cells, and can enhance tumorigenic potential [4,11,50,51]. In addition to glutamine independence, we observed that the cells that had undergone EMT had lower oxygen consumption, lower levels of ATP production, and reduced levels of GSH indicative of reduced mitochondrial activity. Previous work demonstrated that GLS2 mediates mitochondrial activity and GSH, both of which impact tumorigenic potential [6,7,9,10,46,52].

We hypothesized that the loss of GLS2 expression upon the induction of EMT is responsible for the glutamine-independent phenotype and reduced mitochondrial activity. To test this, we over-expressed GLS2 in the GLS2-negative and EMT-enriched breast cancer cell line SUM159. We found that GLS2 over-expression led to an enhancement of mitochondrial activity, glutamine consumption, and GSH and ATP production even in the presence of GLS. Furthermore, this enhanced mitochondrial activity mediated by GLS2 led to a decrease in secreted lactate and reduced the frequency of mammosphere formation. Notably, these effects occurred without altering the expression of EMT markers implying that the metabolic program alone can significantly impact EMT-induced stem-cell properties.

We have previously demonstrated strong evidence that the EMT program imparts stem-cell properties to cancer cells [25,26,28]. EMT is reversible: upon reaching a distant organ, cancer cells turn off the EMT program to proliferate and establish a secondary tumor [53]. Our findings suggest that epithelial-like cancer cells can depend on both glucose and glutamine at the primary site, but upon the activation of the EMT, cancer cells not only acquire migratory and invasive properties but also become glutamine independent. It is reasonable to speculate that the inverse regulation of the two glutaminases provides the metabolic support necessary for the evolving energetic demands during the EMT. Although we observed a reduction in mammosphere formation upon over-expression of GLS2, we did not observe a reduction in expression of EMT transcription factors *FOXC2*, *ZEB1*, or *Snail*. In future studies it would be valuable to test if inhibiting GLS while over-expressing GLS2 confers a more robust attenuation of EMT properties or tumorigenic potential.

We, and others, have established the functional link between EMT and FOXC2 [15,25,26,27,28,30]. Based on these studies, we tested if inhibiting FOXC2 (and thus EMT) would impact GLS2 expression and have metabolic consequences. Indeed, we found that inhibition of FOXC2 expression of activity restored GLS2 expression, mitochondrial activity, and glutamine utilization. Interestingly, GLS expression was not altered by FOXC2 inhibition. These data suggest that the role of GLS and GLS2 is not redundant and that their orchestrated expression provides support for the EMT. In our models, GLS2 expression and EMT features are inversely correlated; therefore, we tested whether GLS2 status was predictive of outcome in breast cancer patients. We found that high levels of *GLS2* are associated with improved survival over 5 years (*p* = 0.019); however, *GLS* levels were not correlated with survival (*p* = 0.045). These findings indicate that the inverse relationship between *GLS2* and *FOXC2* may have a stronger predictive value for outcomes in breast cancer than the correlation between *GLS2* and *GLS* and warrants further validation.

Considerable evidence links the EMT program with the development of resistance to chemotherapy and tumor relapse [54]. For example, triple negative breast cancer tumors with mesenchymal properties are highly resistant to chemotherapies [55]. Our data reveal, for the first time, a GLS2-dependent glutamine metabolic pathway that is influenced by the EMT. Tumors enriched with aggressive and mesenchymal features also exhibit glutamine-independent metabolism suggests that targeting pathways that inhibit the EMT may impact susceptibility to glutaminase inhibitors and is a hypothesis that warrants investigation. Overall, our findings demonstrate that GLS2 has an important role in glutamine metabolism and that re-expression of GLS2 and its downstream metabolism is critical to metastasis.

## 4. Materials and Methods

### 4.1. Cell Culture

All cell lines were cultured at 37 °C in 5% CO_2_. All cell lines derived from HMLE cells were cultured in MEGM (Lonza CC-3051)/DMEM F12 (Mediatech, Wembley, WA, Australia; 10090CV) (1:1) supplemented with penicillin and streptomycin (Gibco/Life Technologies), insulin (Sigma, St. Louis, MO I9278, USA), hEGF (Sigma 9644), hydrocortisone (Sigma H0888), and bovine pituitary extract. MCF7 and MDA-MB-231 cells were cultured in DMEM/F12 (Fisher, Waltham, MA, USA; 10-090-cv) supplemented with 10% fetal bovine serum (FBS) (Sigma), and penicillin and streptomycin. SUM159 cells were cultured in Ham’s F12 (Corning 10-090-cv) medium containing 10% FBS and penicillin and streptomycin. HEK293T cell lines were cultured in DMEM (Corning, Corning, NY, USA; 10-013-CV) supplemented with 10% FBS and penicillin and streptomycin. MCF10A cells were cultured in DMEM/F12 (Corning 10-090-CV) supplemented with 5% horse serum (Sigma H1138), 20 ng/mL human epidermal growth factor (Sigma E9644), 0.5 µg/ml hydrocortisone, 100 ng/mL cholera toxin (Sigma C-8052), 5 µg/mL insulin, and penicillin and streptomycin.

### 4.2. Biolog Assay

Cells were plated at 1 × 10^4^ cells per well into 96-well PM-M2 plates (Biolog, Hayward, CA, USA 13102). Cells were cultured in 50 µL of Biolog M1 medium (Biolog 72301) supplemented with penicillin and streptomycin, 200 mM glutamine, and 5% FBS. After 24 h, 10 µL of Biolog MA redox dye (Biolog 74351) was added to each well, and the plate was placed in the Biolog OmniLog2.3 plate reader to obtain kinetic data at 15 min intervals for 24 h. Data were analyzed with Biolog software packages PMM_Kinetic and PMM_Parametric to obtain the average of the initial rate values at 8 h. Each cell line was tested in triplicate.

### 4.3. Glucose Sensitivity Assay

Cells were plated at 3000 cells per well in 96-well plates in growth medium. After 24 h, medium was removed by aspiration, and cells were washed with PBS. Medium was replenished with 100 µL of glucose and glutamine-free DMEM containing 12 mM, 3 mM, 1.5 mM, 0.8 mM, 0.4 mM, or 0 mM of glucose (Sigma). Each concentration was evaluated in four replicates. After 24 h, 20 µL of CellTiter 96 Aqueous One Solution (Promega, Madison, WI, USA) was added to each well. After incubation at 37 °C for 60 min, absorbance at 490 nm was determined. Raw data were converted to relative percent viabilities, transformed to log, and the IC_50_ was calculated using GraphPad Prism software.

### 4.4. Seahorse Assays

For the MitoStress test, cells were seeded at 15,000 cells per well in an XF96 cell culture plate with the appropriate medium. The XF96 probes were calibrated with 200 µL of calibrant solution and incubated at 37 °C in the absence of CO_2_. After 12 h, the medium was changed to Seahorse base medium (Seahorse Biosciences, Santa Clara, CA, USA) supplemented with 10 mM glucose, 2 mM glutamine, and 1 mM sodium pyruvate and adjusted to pH 7.4. The probe cartridge was prepared to have 0.5 µM oligomycin in port “A”, 0.25 µM FCCP in port “B”, and 0.25 µM Rotenone with 0.25 µM Antimycin in port “C”. For the glycolysis stress test, the probe cartridge was prepared to have 20 µL of 50 mM glucose in port “A”, 11 mM oligomycin in port “B”, and 650 mM 2-deoxyglucose in port “C”. ECAR was normalized to mpH/min. Oxygen consumption rate and extracellular acidification rate were measured by the Seahorse XFe96 Analyzer (Seahorse Biosciences).

### 4.5. ATP Determination

Cells were plated in triplicate at 1 × 10^4^ per well in a 96-well plate. After 24 h, an ATP standard curve was prepared and 100 µL of CellTiter-Glo reagent was added to each test sample and standard. After incubating at room temperature for 10 min, the luminescent signal was recorded with a luminometer.

### 4.6. Glutamate Determination

Samples were analyzed with the Glutamine/Glutamate Determination Kit (Sigma GLN-1) following the manufacturer’s recommendations. Briefly, 5 × 10^5^ cells were plated in triplicate in 6-well plates. After 24 h, growth medium was removed by aspiration, and cells were washed with PBS. Cells were lysed with 250 µL of RIPA buffer. A glutamate standard curve was prepared in a 96-well plate. Test wells contained 25 µL of sample, 175 µL of reaction buffer, and 2 µL of L-GLDH enzyme. The absorbance at 340 nm was measured at time 0 and in 40 min increments until the signal plateaued.

### 4.7. GSH Determination

Cells were plated at 10,000 cells per well in 6 replicates in a 96-well plate. After 24 h, reduced glutathione was assayed with GSH-Glo Glutathione Assay (Promega V6911). Briefly, the growth medium was removed from each well, and cells were washed with PBS. A GSH standard curve was prepared, and cells were incubated with GSH-Glo reagent. After 30 min, Luciferin detection reagent was added to each well and incubated for 15 min. The luminescence was measured using a luminometer.

### 4.8. MitoTracker Red

Cells were plated on glass coverslips at 50% confluency. After 24 h cells were stained with 20 nM MitoTracker Red CMXRos (Life Technologies) under the same culture conditions. After 30 min, cells were fixed with 4% paraformaldehyde. After washing with PBS, coverslips were treated with 5% glycine in PBS for 15 min. Actin was stained with 488 Phalloidin (Alexa Fluor, Waltham, MA, USA), and the nuclei were stained with DAPI. Fluorescent images were obtained with a Zeiss fluorescent microscope. The fluorescence intensities of 100 cells were analyzed and quantified by ImageJ software to obtain the relative fluorescence intensity (RFI).

### 4.9. ^13^C-Glutamine Flux

U-^13^C-Glutamine (17 mg) was added to 50 mL of HMLE medium and filtered. HMLER cells expressing FOXC2 or HMLER control cells were seeded into wells of 10 cm plates at 70% confluency; each cell type was analyzed in four replicates. After 24 h, medium was aspirated, and 5 mL of labeled glutamine solution was added to the cells. A sample was collected immediately after addition. After 6 h, medium was removed by aspiration. Cells were washed with ice-cold PBS and 1 mL of methanol/water (1:1) was added to the cells. Cells were scraped into 15 mL Falcon tubes and immediately flash frozen by submerging tubes into liquid nitrogen. Samples were stored at −80 °C until metabolite extraction. Cell pellets were thawed at 4 °C and subjected to freeze–thaw cycles in liquid nitrogen and ice three times to rupture the cell membrane. Following this, 750 µL of ice-cold methanol/water (4:1) containing 20 µL of internal standards were added to each cell extract. Next, ice-cold chloroform/water (3:1) was added. Organic and aqueous layers were separated. The aqueous portion was deproteinized using a 3-KDa filter (Amicon Ultracel-3K membrane, Millipore Corporation, Burlington, MA, USA), and the filtrate containing metabolites was dried under vacuum (Genevac EZ-2plus, SP Scientific, Warminster, PA, USA). Prior to mass spectrometry, the dried extracts were re-suspended in 100 µL methanol/water (1:1) containing 0.1% formic acid and analyzed by liquid chromatography-mass spectrometry (LC/MS). 

Liquid chromatography was performed using an Agilent 1290 Series HPLC system equipped with a degasser, binary pump, thermostatted auto sampler, and column oven (all from Agilent Technologies, Sana Clara, CA, USA). All samples were kept at 4 °C. A 5 µL aliquot of sample was delivered to a 4.6 mm i.d. × 10 cm Amide XBridge HILIC column (Waters) at 300 µL/min. Chromatography was performed using 0.1% formic acid in water (solvent A) and 0.1% formic acid in acetonitrile (solvent B). Gradients were run starting from 85% solvent B to 30% B from 0–3 min, 30% B to 2% B from 3–12 min, 2% B was held from 12–15 min, 2% B to 85% B from 15–23 min, and 85% B was held for 7 min to re-equilibrate the column.

Samples were analyzed by a 6495 QQQ triple quadrupole mass spectrometer (Agilent Technologies). Metabolites were measured using positive ionization mode with an electrospray ionization (ESI) voltage of 3000 eV. Approximately 9–12 data points were acquired per detected metabolite. Selected reaction monitoring (SRM) was used to determine ^13^C incorporation into glutamine by measuring the expected precursor/product ion pairs: M + 0: 147.08/130.1, M + 1: 148.08/131.1, M + 2: 149.08/132.1, M + 3: 150.08/133.1, M + 4: 151.08/134.1, and M + 5 152.08/135.1. SRM was used to determine ^13^C incorporation into GSH by measuring the expected precursor/product ion pairs: M + 0: 308.0911/76, M + 1: 309.0911/76, M + 2: 310.0911/76, M + 3: 311.0911/76, M + 4: 312.0911/76, and M + 5: 313.0911/76. Mass isotopomer distribution was calculated and corrected for natural abundance.

### 4.10. Steady-State Mass Spectrometry

For profiling of metabolites associated with glycolysis and TCA pathways, 5 × 10^6^ cells were evaluated in triplicate. Cells were collected using trypsin. All cell pellets were stored at −80 °C until analysis. Cell pellets were thawed at 4 °C and subjected to freeze–thaw cycles in liquid nitrogen and ice three times to rupture the cell membrane. Following this, 750 µL of ice-cold methanol/water (4:1) containing 20 µL of internal standards were added to each cell extract. Next, ice-cold chloroform/water (3:1) was added. Organic and aqueous layers were separated. The aqueous portion was deproteinized using a 3-kDa filter (Amicon Ultracel-3K membrane, Millipore Corporation), and the filtrate containing metabolites was dried under vacuum (Genevac EZ-2plus). Prior to mass spectrometry, the dried extracts were re-suspended in 100 µL methanol/water (1:1) containing 0.1% formic acid and analyzed by LC/MS.

Liquid chromatography was performed using an Agilent 1290 Series HPLC system equipped with a degasser, binary pump, thermostatted auto sampler, and column oven (all from Agilent Technologies). All samples were kept at 4 °C. An aliquot of 5 µL of sample was analyzed. Samples were delivered into the spectrometer via normal phase chromatographic separation using a Luna Amino (NH_2_) 4 µm, 100 Å, 2.1 × 150 mm column (Phenominex, Torrance, CA, USA) at 200 µL/min. Chromatography was performed using 5 mM ammonium acetate in water at pH 9.9 (solvent A) and 100% acetonitrile (solvent B). Gradients were run starting from 80% solvent B to 2% B over a 20 min period followed by 2% B to 80% B for a 5 min period, further followed by 80% B for a 13 min period. The flow rate was gradually increased during the separation from 200 µL/min (0–20 min) to 300 µL/min (20.1–25 min) to 350 µL/min (25–30 min) to 400 µL/min (30–37.99 min) and was then returned to 200 µL/min (5 min). Samples were analyzed using a 6495 QQQ triple quadrupole mass spectrometer using SRM. Metabolites associated with glycolysis and TCA pathways were measured using negative ionization mode with ESI voltage of −3500 eV. Approximately 9–12 data points were acquired per detected metabolite.

### 4.11. RNA Extraction and qRT-PCR

Total RNA was isolated using the RNeasy Plus kit (Qiagen, Germantown, MD, USA) according to the manufacturer’s instructions. RNA was quantified with a Thermo Scientific Nanodrop 2000c instrument. RNA sample purity was verified based on the ratio of absorbance at 260 nm to that at 280 nm. RNA samples were normalized to 1 µg. qRT-PCR experiments were run in triplicate, and the mean was used for the determination of mRNA levels. Relative quantification of the mRNA levels was performed using the comparative Ct method with human *RPLPO* as the reference gene and with the formula 2^−ΔΔCt^. The following primer pairs were used: forward RPLPO 5′-GCGACCTGGAAGTCCAACTA-3′ and reverse RPLPO 5′-ATCTGCTTGGAGCCCACAT-3′, forward GLS2 5′-ACACCCTCAGCCTCATGCAT-3′ and reverse GLS2 5′-ATGGCTCCTGATACAGCTGACTT-3′, forward GLS 5′-CACTGCCCTCCCATTACCTAG-3′ and reverse GLS 5′-GAAGCTCAAGCATGGGAACAG-3′, forward FOXC2 5′-GCCTAAGGACCTGGTGAAGC-3′ and reverse FOXC2 5′-TTGACGAAGCACTCGTTGAG-3′, and forward ZEB1 5′-GCACAACCAAGTGCAGAAGA-3′ and reverse ZEB1 5′-CATTTGCAGATTGAGGCTGA-3′.

### 4.12. Immunofluorescence

Cells were plated at 50% confluency on glass coverslips in 6-well plates. After 24 h, the medium was removed aspiration, and cells were washed with PBS. Cells were fixed in 4% paraformaldehyde at room temperature for 20 min and then washed with PBS. Cells were quenched with 4% glycine for 15 min at room temperature and washed with PBS. Cells were blocked with 5% BSA, 0.1% Triton-X100 in PBS for 1 h at room temperature. After three washes with PBS, cells were incubated with (1:200) primary GLS2 (LGA) antibody in 1% BSA for 2 h at room temperature. Cells were washed three times with PBS and then incubated with a secondary antibody at 1:1000 in 1% BSA for 1 h at room temperature in the dark. Cells were washed with PBS and nuclear stain DAPI was added to the cells at 1:1000. After 5 min in the dark. Cells were washed three times with PBS, and coverslips were mounted on glass slides with DAKO mounting solution and incubated at 4 °C overnight to dry. Fluorescent images of the slides were taken with the same exposure time for each slide. The anti-GLS2 was generously provided by Dr. José M. Matés and Dr. Javier Márquez, University of Málaga, and was generated as previously described [18].

### 4.13. Plasmids and Viral Transduction

HMLER-derived cell lines that express Snail and FOXC2, were generated as described previously [42]. The S367E and S367A *FOXC2* mutants were generated as described previously [28]. The HMLE-ZEB1, HMLER-ZEB1, HMLE-Snail/shFOXC2/pZEB1, HMLER-Snail/shFOXC2/pZEB1, and SUM159-shFOXC2/pZEB1 cell lines were made with a lentiviral FUW-2A-mStrawberry ZEB1 expression construct (a gift from Li Ma, MD Anderson, 1515 Holcombe Blvd, Houston, TX 77030, UA) and selected with 4 µg/mL puromycin. For stable GLS2 expression, a GLS2 cDNA expression vector (purchased from MD Anderson core) was subcloned into the pHAGE-EF1alpha-PURO vector (a gift from Kenneth Scott, Baylor College of Medicine, Houston, TX, USA) using the Gateway system (Invitrogen) and selected with 4 µg/mL puromycin. pGIPZ-based shRNAs designed to deplete cells of GLS2 and ZEB1 were purchased from MD Anderson shRNA core.

### 4.14. Breast Cancer Data Analysis

We had previously assembled the EMT-74 data set, a panel of 74 samples across six data sets representing the gene expression profiles of cells before and after an EMT [56]. We examined the gene expression of *GLS* and *GLS2* in the EMT-74 panel samples and compared the expression in the epithelial or mesenchymal state using a Student’s *t*-test. As controls, we evaluated expression of an epithelial marker (*CDH1*), mesenchymal markers (*CDH2*, *VIM*), and a housekeeping gene (*GAPDH*).

### 4.15. Copy Number Analysis

The copy numbers and RNA-seq data for 1075 patients in TCGA was downloaded from the Firehose of the Broad Institute (http://gdac.broadinstitute.org/, January 2016 version). For copy number and RNA expression association analysis, the LinkedOmics portal (http.linkedomics.org) was used. A threshold for copy number data was set by the GISTIC2 algorithm. Primary solid tumors were used for analysis. Statistical analysis was performed using the computing environment R (version 3.4). The one-way analysis of variance (ANOVA) was performed using aov function and *p*-value was obtained. Loss of one or two copies of a gene was considered as a deletion whereas gain in one or two copies of gene was considered as amplification.

### 4.16. L-Lactate Measurement

In a 96 well plate, 1 × 10^4^ cells were plated per well in triplicate. After 24 h, the spent medium was diluted 1:50, and lactate was measured with EnzyChrom L-Lactate Assay Kit (BioAssay Systems, Hayward, CA, USA).

### 4.17. Proliferation Assay

Cells were plated in triplicate at 1 × 10^4^ cells per well in 6 well-plates. Aliquots stained with Trypan Blue were counted manually every 24 h for 72 h. 

### 4.18. Mammosphere Assay

Mammosphere culture medium was made with 1% methylcellulose and MEGM without bovine pituitary extract. In a 96-well low attachment plate, 500 cells per well in six replicates were cultured for 14 days. Every 3 days, 50 µL of fresh media was added. Mammospheres were photographed, and spheres with a diameter greater than 80 µm were counted.

### 4.19. Pan-Cancer Correlation and Survival Analysis

The correlation between expression of *GLS2* and *FOXC2* and EMT score was performed across pan-cancer using the Spearman correlation. The normalized RNA expression data for TCGA samples was downloaded from Broad Firehose release 28 January 2016 run (Firehose, 28 January, 2016 #11383) ([57] https://doi.org/10.7908/C11G0KM9). Primary tumor samples but not normal tissue or metastases samples were selected for analysis. The correlation of the RSEM-normalized RNA expression values between genes in 33 TCGA cancer types was determined using the correlation function cor.test in R (Bioconductor, version 3.4). The EMT score was calculated by single sample gene set enrichment analysis (ssGSEA) using normalized RNA expression of each cancer type and Hallmark gene set (MSigDB database version 6.2) by GSVA package (version 1.3).

Survival analysis was carried out using vital status and overall survival (days from diagnosis to last follow-up) for the TCGA pancancer patients. Only patients with 5 years of follow-up were selected and used for the analysis. Cox proportional-hazards model implemented in *survival* (v2.44) package in R was used for the survival analysis and Cox coefficient was calculated for each cancer type. Visualization was performed using *ggplot2* (v3.2.1) and *survminer* (v0.4.6) packages in R.

### 4.20. Gene Expression Analysis

Gene expression analysis was performed on a previously published dataset as previously described in [43]. Analysis was performed using the ‘limma’ and ‘affy’ packages in R. All *p*-values were adjusted for multiple testing using the Benjamini Hochberg method, significance was set at an adjusted *p*-value < 0.001. Metabolic enzymes and transporters were identified using a previously published comprehensive gene set [58].

### 4.21. Statistical Analysis

Unless otherwise stated, all samples were assayed in triplicate. All in vitro experiments were repeated at least three independent times, and the animal study included 10 mice in each group. Unless otherwise indicated, data are presented as means ± standard deviation (SD), and significance was calculated using the Student’s unpaired two-tailed *t*-test.

### 4.22. Western Blot Analysis and Antibodies

Cell pellets were obtained by trypsinization. Cell pellets were lyzed with RIPA buffer (Sigma R0278) containing phosphatase inhibitor (Roche, Indianapolis, IN, USA; 4906837001) and protease inhibitor (Roche 11697498001) and vortexed vigorously then incubated on ice for 30 minutes, spun down, and cell debris removed. After quantification by Bradford assay, 50 µg of protein was denatured in β-mercaptoethanol at 100 °C for 10 minutes. Samples were loaded onto a 10% SDS-agarose gel and run at 110 V. For blots, antibodies were used at 1:1000 in 5% milk in TBST. Antibodies used were anti-Snail (Cell Signalling, Danvers, MA, USA; 3879S; 1:1000 in BSA), anti-FOXC2 (Bethyl Laboratories, Montgomery, TX, USA; A302–383A; 1:1000 in 5% milk), anti-ZEB1 (Santa Cruz sc-25388; 1:1000 in 5% milk), anti-actin (Santa Cruz, Dallas, TX, USA; sc-1616-R; 1:1000 in 5% milk), and anti-tubulin (Cell Signaling 2144s; 1:1000 in BSA). To quantify the protein band intensity, densitometry analyses were performed by evaluating band intensity of mean gray value using ImageJ software [59]. In brief, the mean gray rectangular areas of the same size were measured in each band. The background was measured above each band. The pixel intensity was inverted for all values by calculating 255 minus the band mean gray value. The background was subtracted and then the protein:loading control ratio was calculated for all samples.

## 5. Conclusions

In conclusion, we demonstrate here that a critical regulator of the EMT, FOXC2, also induces metabolic flexibility in breast cancer. Furthermore, we showed that GLS2 expression and mitochondrial metabolism are altered upon induction of EMT. The expression of GLS2 and mitochondrial metabolic program were restored when FOXC2 expression or activity were inhibited. We also demonstrated that in multiple cancer types, including breast cancer, higher *GLS2* expression relative to *GLS* is a predictor for improved survival and higher GLS2 expression negatively correlates with an EMT signature. Our data provide insights into how metabolic alterations occur in parallel to the onset of the transcriptional program that supports EMT and its aggressive features. Since we found that inhibition of FOXC2 changes the metabolism of cancer cells by promoting mitochondrial activity, future work is warranted to test whether combination treatment strategies can be employed to take advantage of altered metabolic states in cells that have features of EMT.

## Supplementary Materials

The following are available online at https://www.mdpi.com/2072-6694/11/10/1610/s1, Figure S1: GLS and GLS2 are inversely associated in multiple models of EMT-induced cell lines; Figure S2: Cells induced to undergo EMT exhibit metabolic reprogramming; Figure S3: FOXC2 expression does not change after GLS2 over-expression; Figure S4: Protein expression of Zeb1, FOXC2, Snail, Tubulin, and Actin in HMLER cell lines; Figure S5: Expression of GLS2 inversely correlated with FOXC2
