# Supplementary material for: The Epithelial to Mesenchymal Transition Promotes Glutamine Independence by Suppressing GLS2 Expression
Source: Cancers (Basel). 2019 Oct 22;11(10):1610. doi: 10.3390/cancers11101610 (PMC6826439; doi:10.3390/cancers11101610)
Supplement: Supplementary file 1 [file cancers-11-01610-s001.pdf]

Supplementary Materials

The Epithelial to Mesenchymal Transition Promotes Glutamine Independence by Suppressing *GLS2* Expression

Esmeralda Ramirez-Peña, James Arnold, Vinita Shivakumar, Robiya Joseph, Geraldine Vidhya Vijay, Petra den Hollander, Neeraja Bhangre, Paul Allegakoen, Rishika Prasad, Zachary Conley, José M. Matés, Javier Márquez, Jeffrey T. Chang, Suhas Vasaikar, Rama Soundararajan, Arun Sreekumar and Sendurai A. Mani

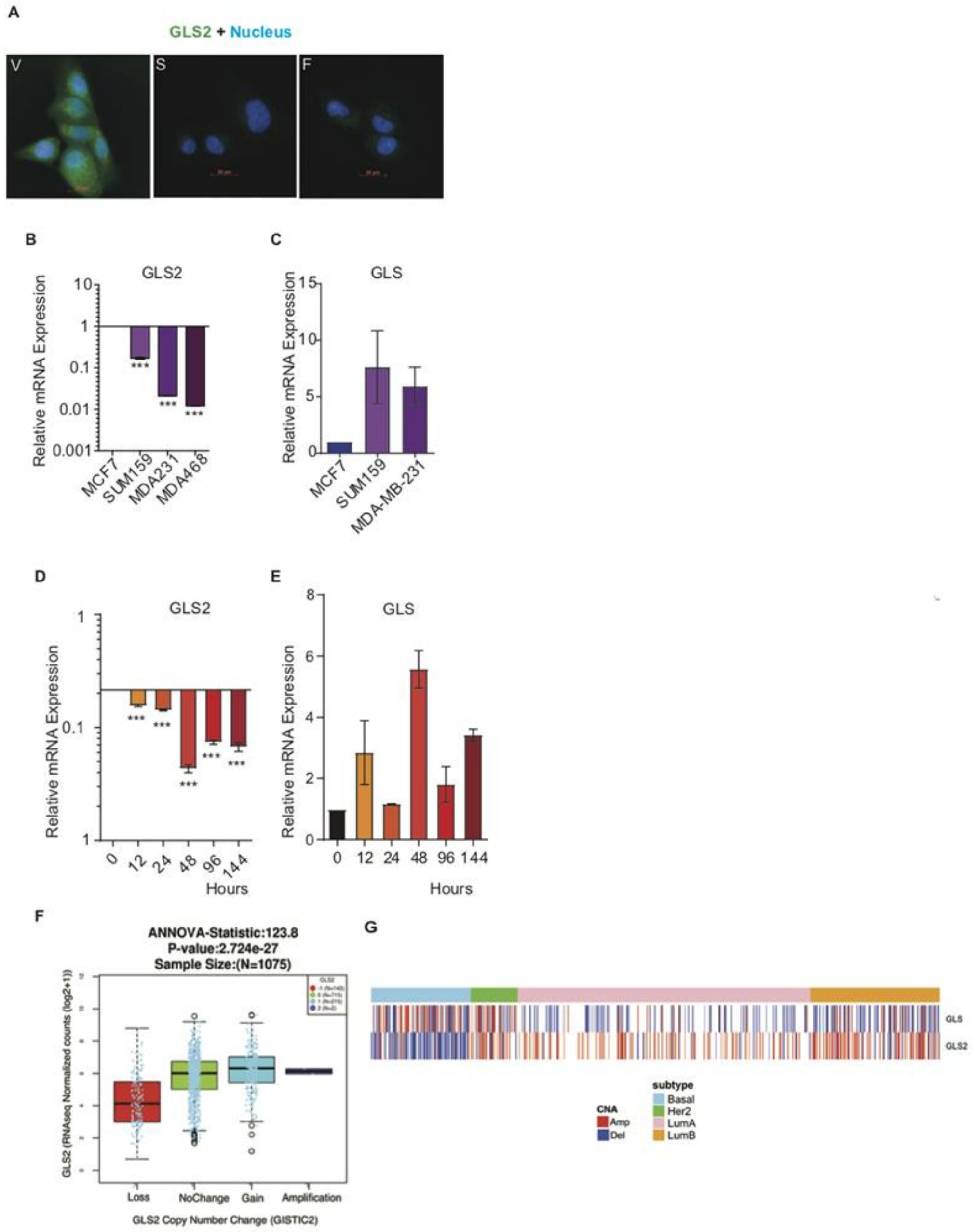

Figure S1. GLS and GLS2 are inversely associated in multiple models of EMT-induced cell lines. (**A**) Representative immunofluorescent images of HMLER-vector (V), HMLER-SNAIL (S), and HMLER-FOXC2 (F) cells stained with human GLS2 antibody (green) co-stained with Dapi (blue). Scale bar: 20

μm. (**B**) GLS2 mRNA expression in MCF7 (*n* = 3), SUM159 (*n* = 3), MDA231 (*n* = 3), and MDA468 (*n* = 3) cells. (**C**) RT-PCR of GLS mRNA in MCF7, SUM159, and MDA-MB231 (*n* = 3). (**D**) GLS2 mRNA levels in MCF10A cells treated with 5 ng/mL of TGFβ for 12, 24, 48, 96, and 144 hours (*n* = 3). (**E**) RT-PCR of GLS mRNA in MCF10A cells treated with 5 ng/mL of TGFβ for 0, 12, 24, 48, 96, and 144 hours (*n* = 3). (**F**) Copy number change of GLS2 in breast cancer patient samples. Deletion in one copy (-1) of GLS2 gene is observed in 143 patients in the TCGA Breast cancer cohort. (**G**) Amplifications (red, copy number gain, +1 and amplification, +2) and deletions (blue, copy number loss, −1 and deletion, −2) of GLS and GLS2 by PAM50 subtype.The data are reported as means +/− SD; NS indicates $p > 0.05$, * $p \leq 0.05$, ** $p \leq 0.01$, *** $p \leq 0.001$.

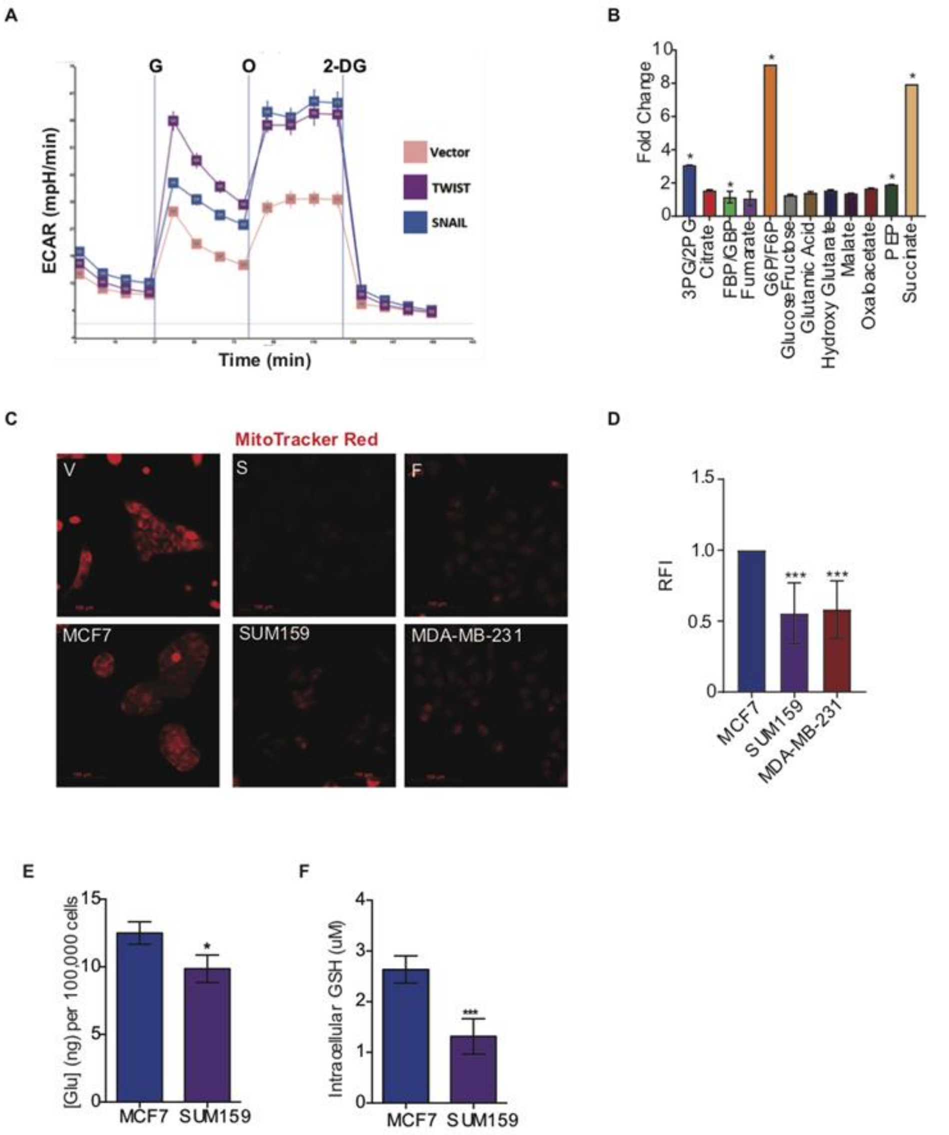

Figure S2. Cells induced to undergo EMT exhibit metabolic reprogramming. (**A**) Extracellular acidification rate (ECAR) over time with the addition of glucose (G), oligomycin (O), and 2-deoxyglucose (2-DG) in HMLE-SNAIL (*n* = 5) and HMLE-TWIST (*n* = 5) cells compared to epithelial HMLE-GFP (*n* = 5) control cells measured using the Seahorse XFe96 Analyzer. (**B**) TCA cycle and glycolysis metabolites were quantified by mass spectrometry in HMLER-FOXC2 (*n* = 3) and HMLER-vector (*n* = 4) cells. Plotted is the fold change. (**C**) Representative immunofluorescent images taken at 20× magnification of MitoTracker Red (red) staining of HMLER-vector, HMLER-SNAIL, HMLER-FOXC2, and breast cancer cell lines MCF7, SUM159, and MDA-MB-231. (**D**) Quantification of relative fluorescence intensity (RFI) from MitoTracker Red staining in MCF7 (*n* = 100), SUM159 (*n* = 100), and MDA-MB-231 (*n* = 100) cells calculated with ImageJ software. (**E**) Quantification of intracellular

glutamate in MCF7 (n = 3) and SUM159 (n = 3) cells. (**F**) Quantification of intracellular GSH in MCF7 (n = 5) and SUM159 cells (n = 5). The data are reported as means +/− SD; NS indicates $p > 0.05$, * $p \leq 0.05$, ** $p \leq 0.01$, *** $p \leq 0.001$.

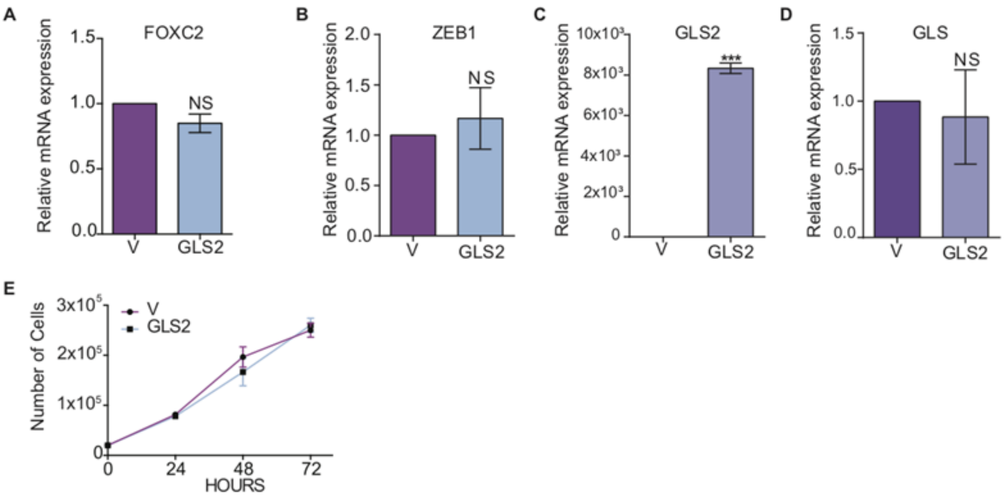

Figure S3. FOXC2 expression does not change after GLS2 over-expression. (**A**)RT-PCR of FOXC2 mRNA in SUM159-vector (V) and SUM159 over-expressing GLS2 (GLS2) (n = 3). (**B**) RT-PCR analysis of ZEB1 mRNA in SUM159-vector (V) and SUM159-GLS2 (GLS2) cells (n = 3). (**C**)RT-PCR analysis of GLS2 mRNA in HMLER-SNAIL-vector (V) and HMLER-SNAIL-GLS2 (GLS2) cells (n = 3). (**D**) RT-PCR analysis of GLS mRNA in HMLER-SNAIL-vector (V) and HMLER-SNAIL-GLS2 (GLS2) cells (n = 3). (**E**) Proliferation measured by number of cells counted at 0, 24, 48, 72 hours (n = 3). The data are reported as mean +/− SD (NS $p > 0.05$, * $p \leq 0.05$, ** $p \leq 0.01$, *** $p \leq 0.001$).

A

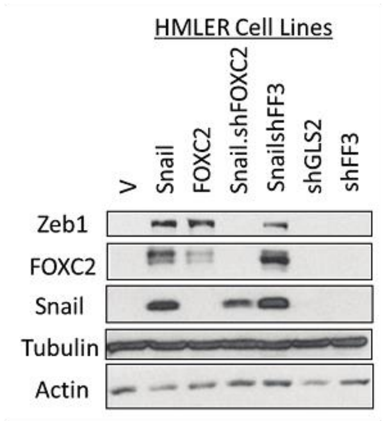

B

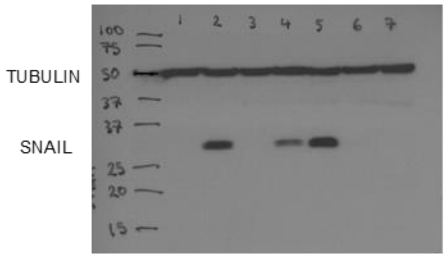

C

LANE	SNAIL	TUBULIN	RATIO
1	15.61	163.63	0.10
2	146.59	158.73	0.92
3	10.99	158.69	0.07
4	86.36	168.72	0.51
5	163.66	165.19	0.99
6	11.45	156.52	0.07
7	6.61	158.36	0.04

D

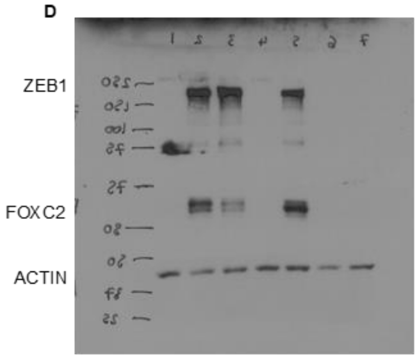

E

LANE	FOXC2	ACTIN	RATIO
1	1.17	36.88	0.03
2	63.39	19.84	3.20
3	25.89	30.35	0.85
4	0.83	33.91	0.02
5	75.86	38.45	1.97
6	-0.04	17.06	0.00
7	0.13	32.21	0.00

F

LANE	ZEB1	ACTIN	RATIO
1	-0.83	36.88	-0.02
2	78.80	19.84	3.97
3	85.01	30.35	2.80
4	-0.82	33.91	-0.02
5	62.00	38.45	1.61
6	-0.77	17.06	-0.04
7	-0.82	32.21	-0.03

Figure S4. Protein expression of Zeb1, FOXC2, Snail, Tubulin, and Actin in HMLER cell lines. (**A**) Western blot of HMLER-V, HMLER-Snail, HMLER-FOXC2, HMLER-SNAIL-shFOXC2, HMLER-SNAIL-shFF3 (vector control), HMLERshGLS2, and HMLERshFF3 (vector control) (**B**) Scan of full blot with Tubulin and Snail proteins (**C**) Quantification of relative Snail and tubulin protein levels and the protein: loading control ratio analyzed by ImageJ. (**D**) Scan of full blot with ZEB1, FOXC2 and Actin proteins (**E**) Quantification of relative FOXC2 and Actin protein levels and the protein: loading control ratio analyzed by ImageJ. (**F**) Quantification of relative ZEB1 and Actin protein levels and the protein: loading control ratio analyzed by ImageJ.

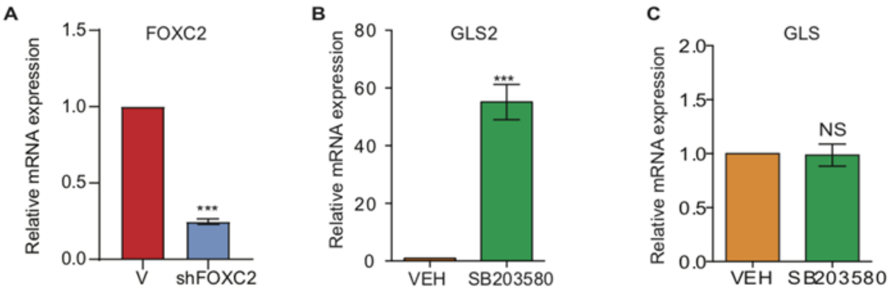

Figure S5. Expression of GLS2 inversely correlated with FOXC2. (**A**) RT-PCR analysis of FOXC2 mRNA expression in HMLER-SNAIL-vector (V) and HMLER-SNAILshFOXC2 (shFOXC2) cells (n = 3). (**B**) RT-PCR analysis of GLS2 mRNA expression in HMLER-FOXC2 cells treated with vehicle (VEH) or with 20 μM SB203580 for 24 hours (n = 3). (**C**) RT-PCR analysis of GLS mRNA expression in HMLER-FOXC2 cells with vehicle and SB203580 treatment for 24 hours (n = 3). The data are reported as mean +/− SD (NS $p > 0.05$, * $p \leq 0.05$, ** $p \leq 0.01$, *** $p \leq 0.001$).

© 2019 by the authors. Licensee MDPI, Basel, Switzerland. This article is an open access article distributed under the terms and conditions of the Creative Commons Attribution (CC BY) license (http://creativecommons.org/licenses/by/4.0/).
